# The Pattern of Highly Pathogenic Avian Influenza H5N1 Outbreaks in South Asia

**DOI:** 10.3390/tropicalmed4040138

**Published:** 2019-11-27

**Authors:** Sukanta Chowdhury, Mohammad Enayet Hossain, Probir Kumar Ghosh, Sumon Ghosh, Muhammad Belal Hossain, Cindy Beard, Mahmudur Rahman, Mohammed Ziaur Rahman

**Affiliations:** 1 International Centre for Diarrhoeal Disease Research, Bangladesh (ICDDR,B), Dhaka 1212, Bangladesh; enayet.hossain@icddrb.org (M.E.H.); probir@icddrb.org (P.K.G.); sumon.ghosh@icddrb.org (S.G.); belal.hossain@icddrb.org (M.B.H.); rahman.mahmudur@icddrb.org (M.R.); mzrahman@icddrb.org (M.Z.R.); 2 Department of Epidemiology, Fielding School of Public Health, University of California, Los Angeles, CA 90095, USA; cmbeard92@gmail.com

**Keywords:** highly pathogenic avian influenza viruses, H5N1, poultry, human, South Asia

## Abstract

Highly pathogenic avian influenza (HPAI) H5N1 has caused severe illnesses in poultry and in humans. More than 15,000 outbreaks in domestic birds from 2005 to 2018 and 861 human cases from 2003 to 2019 were reported across the world to OIE (Office International des Epizooties) and WHO (World Health Organization), respectively. We reviewed and summarized the spatial and temporal distribution of HPAI outbreaks in South Asia. During January 2006 to June 2019, a total of 1063 H5N1 outbreaks in birds and 12 human cases for H5N1 infection were reported to OIE and WHO, respectively. H5N1 outbreaks were detected more in the winter season than the summer season (RR 5.11, 95% CI: 4.28–6.1). Commercial poultry were three times more likely to be infected with H5N1 than backyard poultry (RR 3.47, 95% CI: 2.99–4.01). The highest number of H5N1 outbreaks was reported in 2008, and the smallest numbers were reported in 2014 and 2015. Multiple subtypes of avian influenza viruses and multiple clades of H5N1 virus were detected. Early detection and reporting of HPAI viruses are needed to control and eliminate HPAI in South Asia.

## 1. Introduction

Highly pathogenic avian influenza (HPAI) H5N1 has caused a large number of outbreaks in poultry in Asia, Europe, and Africa [1]. Chickens are susceptible to HPAI (H5N1), with high morbidity and a case fatality rate as high as 100% [2]. Wild birds including shorebirds and gulls and domestic ducks are considered to be the natural reservoir of the virus. These animals may carry and shed HPAI viruses without showing any signs of illness [3,4,5]. Thus they become silent carriers, sustaining and perpetuating H5N1, and transmitting the virus to other susceptible hosts [3,6]. 

A total of 68 countries reported HPAI outbreaks to the World Organization for Animal Health (OIE) between January 2003 and January 2018 [7]. Globally, more than 15,000 outbreaks were reported in domestic birds from January 2005 to January 2018 [7]. In 2003, HPAI (H5N1) reemerged in both poultry and humans in Southeast Asian countries [8,9]. Domestic poultry from East and Southeast Asian countries have been infected most frequently, and H5N1 became endemic in domestic poultry in these countries. Thus, it has caused severe economic losses. As of December 2006, more than 240 million poultry including chicken, ducks, turkeys, and geese died or had been culled to prevent the spread of H5N1 [10].

Since 2003, 17 countries have reported a total of 861 human cases ofH5N1 to the World Health Organization (WHO), with most cases arising in Asian countries. The global case fatality rate among all these cases was >50% [9]. Epidemiological studies have found that most human cases of H5N1 infection involved the subject having a history of poultry exposure such as slaughtering and/or consuming sick poultry and handling infected live and dead poultry [11,12,13,14]. 

In South Asia, people live very close to their domestic poultry and have limited knowledge about the risk of avian influenza. Among the eight South Asian countries, six countries (Afghanistan, Bangladesh, Bhutan, India, Nepal, and Pakistan) reported HPAI (H5N1) outbreaks in poultry since 2006, and two countries (Bangladesh and Pakistan) reported human cases of H5N1 infection since 2007. The Maldives and Sri Lanka did not report any HPAI outbreaks in poultry or in humans [1,9]. Although there have been few human cases in South Asian countries, consistent circulation of H5N1 in poultry and seasonal influenza viruses in humans can promote genetic reassortment and evolution of novel virus strains with the potential for public health importance [15]. Substantial knowledge about the spatial and temporal distribution of highly pathogenic avian influenza virus in South Asian countries is essential to develop a strategic plan for preventing and controlling future outbreaks of HPAI. In this study, we described the spatial and temporal distribution of HPAI outbreaks in South Asia from January 2006 to June 2019. The findings of this study will help to identify times, places, and hosts most associated with a high risk of H5N1 infection in South Asia.

## 2. Methods

### 2.1. Sources of HPAI Outbreak Data

We extracted data for all reported HPAI outbreaks in poultry between January 2006 and June 2019 from the OIE website. We considered the starting date of each outbreak as the onset month of the outbreak [7]. We organized the extracted data by year and month to estimate the average peak month for HPAI infections in the South Asian countries (Afghanistan, Bangladesh, Bhutan, India, Nepal, Pakistan, Maldives, and Sri Lanka). Data about human cases of H5N1 were obtained from the WHO website [9].

### 2.2. Epidemiological and Microbiological Data

We collected data on the epidemiology of H5N1 and on the phylogenetics of virus isolates from published articles. Data from different South Asian countries were compared to determine the similarities and dissimilarities. We performed an internet-based systematic search in Google Scholar and PubMed to collect data from published articles using the following keywords—“avian influenza”, “HPAI”, “H5N1”, “avian influenza in Afghanistan”, “avian influenza in Bangladesh”, “avian influenza in India”, “avian influenza in Pakistan”, “avian influenza in Bhutan”, “avian influenza in Nepal”, “avian influenza in Sri Lanka”, “avian influenza in Maldives”, “risk factors for avian influenza”, “avian influenza in poultry”, “avian influenza in humans”, and “H5N1 clades”.

### 2.3. Time Periods

In this study, we used H5N1 outbreak data between January 2006 and June 2019. We also collected molecular data on avian influenza viruses from published articles and abstracts. All articles and abstracts reviewed were published between 1st January 2006 and June 2019. We identified a total of 50 articles and abstracts for our analysis that were relevant to our research interest. 

### 2.4. Statistical Analysis

We performed descriptive analyses to show the total numbers of outbreaks, geographic distributions, affected host species, husbandry practices of affected birds, and peak month of H5N1 outbreak detection. For each country, we determined the average month during which the highest number of H5N1 outbreaks were reported to the OIE. We estimated the relative risk (RR) with 95% confidence interval (CI) to identify the association between H5N1 outbreak occurrence and exposure variables using Poisson regression.

### 2.5. Mapping the H5N1 Outbreak in Poultry and in Humans

We used latitude and longitude data of reported H5N1 outbreaks in poultry and in humans in the countries of interest using Google Earth software. The latitude and longitude data for each reported H5N1 outbreak were collected from the World Organization for Animal Health (OIE) and the World Health Organization (WHO) websites.

### 2.6. Phylogenetic Analysis

A total of 46 H5N1 virus isolates were selected from Afghanistan, Bangladesh, Bhutan India, and Nepal for phylogenetic analysis. Nucleotide sequences of the HA (haemagglutinin) gene of these selected H5N1 isolates were downloaded from GenBank. The HA gene sequences of the selected isolates were subjected to ClustalW multiple sequence alignment using the BioEdit 7.2 program [16]. A phylogenetic tree was constructed with the Kimura 2-parameter model and neighbor-joining methods (with 1000 bootstrap replications) using MEGA 7 [17].

## 3. Results

### 3.1. Descriptive Epidemiology of H5N1 Outbreaks in Poultry and Birds

**Figure 1:**
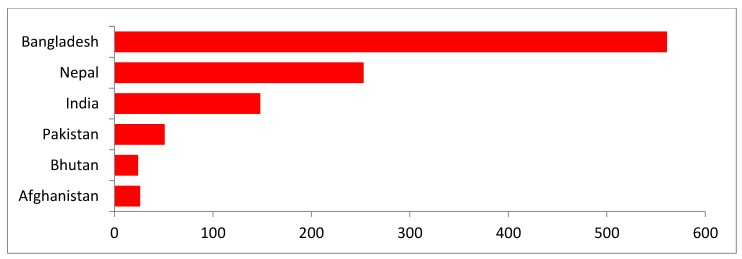
Reported H5N1 outbreaks in South Asia by country, January 2006 to June 2019.

**Figure 2:**
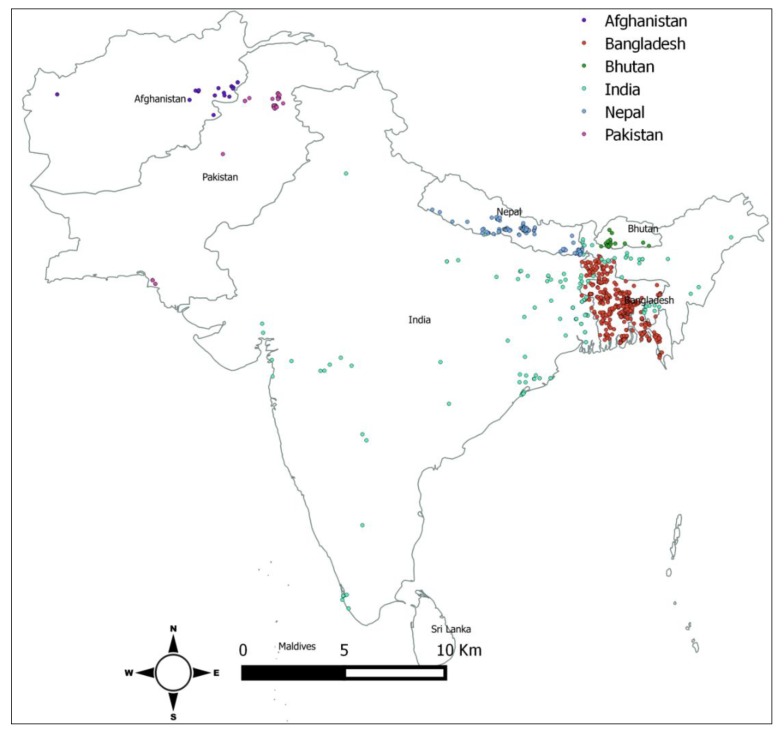
Spatial distribution of H5N1 outbreaks in South Asia, January 2006 to June 2019.

Out of the eight South Asian countries, six countries reported HPAI in poultry and wild birds during the time period of interest. To date, Maldives and Sri Lanka have not reported any H5N1 outbreaks. Between January 2006 and June 2019, a total of 1063 H5N1 outbreaks were reported to OIE by South Asian countries (Figure 1 and Figure 2). Bangladesh reported the highest number (n = 561) of H5N1 outbreaks in poultry and wild birds from January 2006 to June 2019. Afghanistan, India, and Pakistan first reported H5N1 outbreaks in 2006. Bangladesh reported its first outbreak in 2007, followed by Nepal in 2009, and Bhutan in 2010 (Table 1). Among all the reported H5N1 outbreaks, 97% were reported in domestic poultry. Commercial poultry were three times more likely to be infected with H5N1 than backyard poultry (RR 3.47, 95% CI: 2.99–4.01) (Table 2).

### 3.2. Temporal Patterns of H5N1 Outbreaks in Poultry and Birds

**Figure 3:**
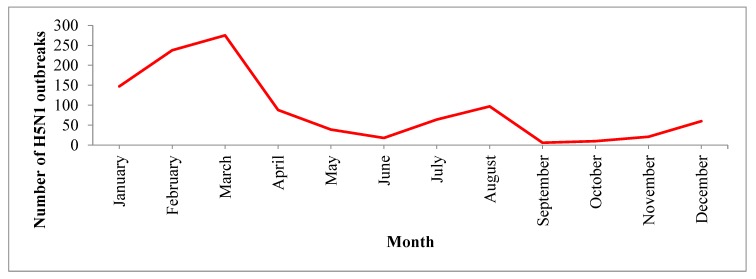
Distribution of H5N1 outbreaks in poultry in South Asian countries by month, January 2006 to June 2019.

**Figure 4:**
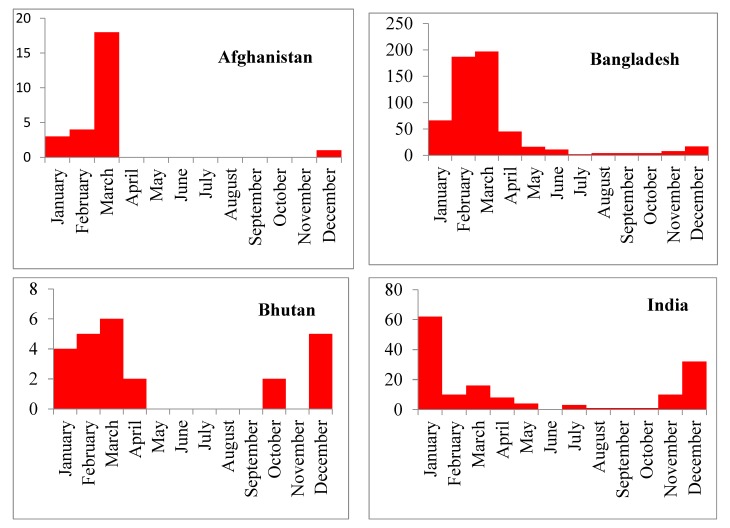

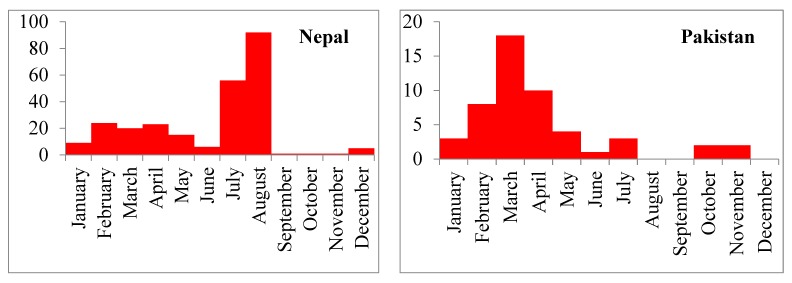
Country and month-wise H5N1 outbreaks in poultry and birds in South Asia, January 2006 to June 2019 (for six countries separately). The numbers given in y-axis correspond to the number of H5N1 outbreaks.

**Figure 5:**
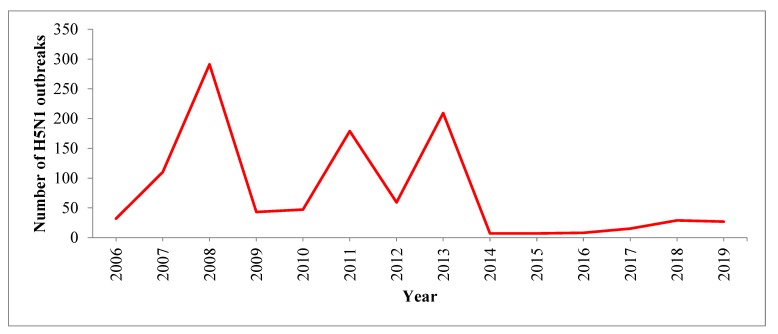
Distribution ofH5N1 outbreaks in poultry and birds in South Asian countries by year, January 2006 to June 2019.

In South Asia, most H5N1 outbreaks (741/1063, 70%) were reported during the winter season (November to March). H5N1 outbreaks were reported most frequently in March (Figure 3). Figure 4 provides country-wise distributions of reported H5N1 outbreaks by month. The highest numbers of H5N1 outbreaks were reported in 2008 and the smallest numbers of H5N1 outbreaks were reported in 2014 and 2015 (Table 3 and Figure 5). In this region, India reported the first H5N1 outbreak in poultry on 27 January 2006 and Nepal reported the last H5N1 outbreak on 18 June 2019.

### 3.3. Distribution of H5N1 Clades in Poultry and Birds

**Figure 6:**
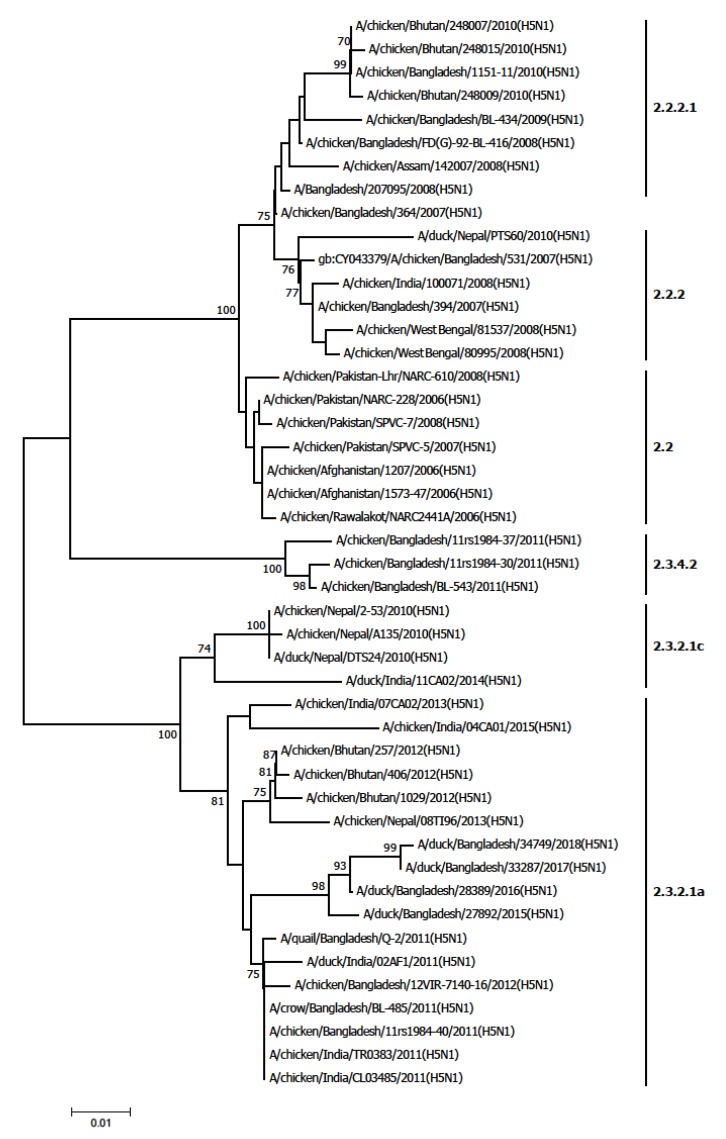
Phylogenetic tree based on the HA gene of H5N1 viruses in poultry in South Asia.

Between January 2003 and December 2018, multiple clades of the H5N1 virus were identified in poultry across South Asia (Table 4 and Figure 6). Clade diversification was mostly observed in Bangladesh and India. The most commonly identified clade was 2.2. So far, two different clades have been reported in Afghanistan, eight clades in Bangladesh, five clades in Bhutan, eight clades in India, three clades in Nepal, and two clades in Pakistan. The most recently detected clade ofH5N1 virus in this region was 2.3.2.1a. In South Asia, clade 2.2 was introduced first in poultry which has been replaced by other clades over the period. In 2010, a new clade 2.3.2.1 appeared in Nepal as the first South Asian country, which later identified in Bangladesh, India, and Bhutan causing a massive outbreak in poultry.

### 3.4. Other Reported Subtypes of Avian Influenza in Poultry and Birds

In South Asia, many other subtypes of HPAI and low pathogenic avian influenza (LPAI) viruses were also detected during the time period of interest. A total of 31 subtypes were identified in the published literature (Table 5). The most prevalent subtype of HPAI viruses was H5N1. The LPAI H9N2 subtype was also common. Very recently, Bangladesh reported the H5N6 subtype in domestic waterfowl. The most diversity of avian influenza viruses in poultry was observed in Bangladesh.

### 3.5. Human Cases

In South Asia, only 12 total cases of human H5N1 infections had been reported to the WHO as of 24 June 2019 (Table 6). Only Bangladesh, Pakistan, and Nepal have reported human cases of the virus. Bangladesh reported the highest number of human cases (n = 8) during the period of interest. Pakistan reported the first human case in the region in 2007. Among these 12 human cases, three were fatal. Clade 2.2 viruses caused infection in humans in Bangladesh and Pakistan during 2007 to 2008. In 2011, clade 2.2.2 was detected in humans in Bangladesh. In 2012, clade 2.3.2.1 was isolated from three human cases in Bangladesh. During 2015 to 2019, a new clade 2.3.2.1a viruses were detected in humans in Bangladesh and Nepal [43].

## 4. Discussion

This study provides an update on the state of avian influenza in South Asia. H5N1 outbreaks in this region repeatedly occurred in poultry between January 2006 and June 2019. However, human cases of H5N1 infection were sporadic in nature. According to the OIE, a total of 7122 HPAI outbreaks were reported in domestic birds across 68 countries from January 2013 to August 2018. Asia, Africa and Europe were the regions most affected by these outbreaks [7]. Between January 2006 and June 2019, South Asian countries reported more than one thousand H5N1 outbreaks to OIE. South Asia detected the first H5N1 outbreak in poultry in 2006, with the highest number of outbreaks in the region reported in 2008. Though few H5N1 outbreaks were reported after 2014, circulation of H5N1 viruses continues. Findings from surveillance and other studies suggest that both HPAI and LPAI viruses continue to circulate among birds in South Asian countries [20,21,27,30,33,35,37,42,43]. 

In this analysis, we found that HPAI (H5N1) outbreaks in the region occurred most frequently in the winter season (January to March), with the largest number of outbreaks occurring specifically in March. This finding is similar to the results from Southeast Asian countries where H5N1 outbreaks were frequently detected during winter in poultry [44]. HPAI (H5N1) outbreaks globally showed a clear seasonal pattern in poultry, humans and wild birds; most human cases (50%) were reported during January to March [15]. This seasonality could be due to the lower ambient temperature (average minimum temperature 13.9 °C). Lower temperatures are suitable for the survival of avian influenza viruses. Avian influenza viruses persist in cold water for a long time and this may be associated with a higher chance of transmission [45]. One study suggested that lower ambient temperatures may cause decreased immunity in poultry and thus make the birds more susceptible to infection with the H5N1 virus [15]. The circulation of avian influenza viruses is also related to the migration of wild birds, particularly during the winter season, as reported by some global studies [18,46,47].

This analysis noted that the high incidence of H5N1 outbreak in commercial poultry farms in Bangladesh, Nepal and Pakistan. India, Afghanistan and Bhutan reported more outbreaks in backyard poultry flock. This geographical diversity for H5N1 outbreaks within South Asia could be due to the variation in farm biosecurity system, proximity to the migratory bird flyway, wild bird-domestic poultry interaction, animal health infrastructure and strength of outbreak reporting system. 

Avian influenza viruses continue to evolve and infect a wide range of poultry species and humans. In South Asia, multiple clades of the H5N1 virus were detected in poultry from 2006 to 2019. The most commonly detected clade in this region was 2.2. Clade 2.3.2.1a was also common and was identified in India, Bangladesh, Nepal and Bhutan [18,19,20]. Clade 2.3.2.1a was detected in India, Bangladesh, Nepal and Bhutan which shared common ancestry [23]. India identified clade 2.2 during outbreaks in 2006–2007, clade 2.2.2.1 in 2008–2010, clade 2.3.2.1a in 2011-2015 and clade 2.3.2.1c during in 2014–2015 [21,22,23]. Bangladesh identified clade 2.2 during outbreaks in 2007–2010, clade 2.3.4 in 2011, clade 2.3.2 in 2011, clade 2.3.2.1 in 2011 and clade 2.3.2.1a in 2014 [18,24]. The co-detection of genetically related avian influenza viruses in human and poultry during the same period suggested that viruses were transmitted from poultry to human in Bangladesh Pakistan and Nepal.

Multiple subtypes of avian influenza viruses were identified in South Asian countries between January 2006 and June 2019, including: H5N1, H5N6, H5N2, H5N3, H9N2, H1N1, H1N2, H1N3, H1N4, H2N3, H2N4, H3N1, H3N2, H3N5, H3N6, H3N8, H4N1, H4N2, H4N6, H5N8, H6N1, H6N2, H7N3, H6N7, H7N9, H10N7, H11N1, H11N2, H11N6, H11N9 and H11N3 [23,29,34,35,36,38,39,40,41,42,43]. Avian influenza strain diversity may increase the probability of genetic reassortment between influenza subtypes which may facilitate the evolution of a future novel pathogenic strain of animal and public health importance. Similar viral diversity was also observed in other Asian countries [48,49].

Human cases of H5N1 were few despite high numbers of avian influenza outbreaks among birds in South Asia. This finding was unusual compared to other avian influenza affected countries like China, Egypt, Indonesia, Thailand and Vietnam, which reported a large number of human cases to the WHO during the period of interest [9]. The mortality rate for human H5N1 infections in South Asia (25%) was lower than that observed globally (>50%) [9]. The majority of human cases in the region had a history of poultry exposure [50,51]. Though human cases of HPAI were rare, continuous circulation of the H5N1 virus in the poultry population continues to provide potential opportunities for transmission to humans. 

## 5. Conclusions

This analysis provides an overall update on HPAI (H5N1) outbreaks in South Asia. The emergence of diverse genetic clades of H5N1 viruses in poultry in South Asia indicates the continuing evolution of HPAI viruses with the potential to become of public health interest. Despite the low number of reported human cases, people in this region are highly vulnerable to HPAI viruses due to their close and frequent interaction with poultry. Continuous monitoring is necessary to identify both existing and novel avian influenza viral strains circulating in poultry, wildlife and humans using a One Health approach. Individual country experiences on poultry production system, farm biosecurity, and outbreak reporting system may be useful to develop and design avian influenza control strategies. Strong regional collaboration and cooperation is essential for pandemic influenza preparedness planning and response in South Asia.

## Figures and Tables

**Table 1:** Summary of reported H5N1 outbreaks among poultry, wild birds, and humans in South Asian countries, January 2006 to June 2019.

Country	H5N1 Outbreaks in Poultry and Wild Birds			Total Reported Human Cases ofH5N1
	Number of Outbreaks	Date of Initial Outbreak	Date of Last Outbreak	
Afghanistan	26	6 March 2006	23 January 2018	No reported cases
Bangladesh	561	5 February 2007	15 December 2018	8
Bhutan	24	18 February 2010	6 April 2019	No reported cases
India	148	27 January 2006	23 March 2019	No reported cases
Maldives	-	No reported outbreak	-	No reported cases
Nepal	253	8 January 2009	18 June 2019	1
Pakistan	51	23 February 2006	17 June 2008	3
Sri Lanka	-	No reported outbreak	-	No reported cases

**Table 2:** Country-wise characteristics of H5N1 outbreaks by host species, husbandry practices and seasonality.

Variables	Afghanistan	Bangladesh	Bhutan	India	Maldives	Nepal	Pakistan	Sri Lanka	Total (%)	* RR (95% CI)
**Species**										
Wild birds	2	6	-	20	-	4	2	-	34 (3)	Ref.
Domestic poultry	24	555	24	128	-	249	49	-	1029 (97)	30.26 (21.5–42.6)
**Husbandry practices**										
Backyard farm	21	45	20	100	-	40	4	-	230 (22)	Ref.
Commercial farm	3	510	4	28	-	208	44	-	797 (75)	3.47 (2.99–4.01)
Wild	2	6	-	20	-	5	3	-	36 (3)	0.15 (0.11–0.22)
**Season**										
Summer (April–June)	0	72	2	12	-	44	15	-	145 (14)	Ref.
Monsoon (July–October)	0	14	2	6	-	150	5	-	177 (17)	1.2 (0.98–1.52)
Winter (November–March)	26	475	20	130	-	59	31	-	741 (70)	5.11 (4.28–6.1)

* RR = Relative risk.

**Table 3:** Year-wise H5N1 outbreaks by country, South Asia, January 2006 to June 2019.

Year	Afghanistan	Bangladesh	Bhutan	India	Nepal	Pakistan	Total
2006	13	0	0	7	0	12	32
2007	9	68	0	1	0	32	110
2008	0	225	0	59	0	7	291
2009	0	31	0	10	2	0	43
2010	0	30	5	5	7	0	47
2011	0	170	4	4	1	0	179
2012	0	23	9	8	19	0	59
2013	0	2	1	3	203	0	209
2014	0	0	0	6	1	0	7
2015	0	0	1	6	0	0	7
2016	0	1	1	6	0	0	8
2017	1	8	0	3	3	0	15
2018	3	3	2	18	3	0	29
2019	0	0	1	12	14	0	27
Total	26	561	24	148	253	51	1063

**Table 4:** H5N1 viral clades reported in poultry in South Asian countries, January 2003 to December 2018 [18,19,20,21,22,23,24,25,26,27,28,29,30,31,32,33].

Countries	Clades (Year of Detection)	Host Species
Afghanistan	2.2 (2006), 2.2.3 (2006)	Chicken
Bangladesh	2.2 (2007), 2.2.2 (2007), 2.2.3 (2007), 2.3.2 (2009), 2.3.4 (2011), 2.3.2.1 (2011), 2.3.2.1a (2015), 2.3.4.2 (2011)	Chicken, duck, geese, turkey, quail
Bhutan	2.2 (2010), 2.2.2 (2010), 2.2.3 (2010), 2.3.2.1 (2012) 2.3.2.1a (2012)	Chicken
India	2.2 (2006), 2.2.2 (2008), 2.2.3 (2008), 2.3.2 (2011), 2.2.2.1 (2008), 2.3.2.1 (2011), 2.3.2.1a (2011), 2.3.2.1c (2014)	Chicken, duck, turkey
Maldives	-	-
Nepal	2.2 (2010), 2.3.2 (2010), 2.3.2.1 (2010)	Chicken, duck
Pakistan	2.2 (2006), 2.2.3 (2006)	Chicken
Sri Lanka	-	-

**Table 5:** Distribution of avian influenza viruses subtypes in poultry in South Asia, January 2003 to December 2018 [23,34,35,36,37,38,39,40,41,42,43].

Countries	Subtypes	Host Species
Afghanistan	H5N1	Chicken, turkey
Bangladesh	H5N1, H5N6, H5N2, H5N3, H9N2, H1N1, H1N2, H1N3, H1N4, H2N3, H2N4, H3N2, H3N5, H3N6, H3N8, H4N1, H4N2, H4N6, H6N1, H6N2, H6N7, H7N9, H10N7, H11N1, H11N2, H11N6, H11N9, H11N3	Chicken, duck, geese, turkey, quail
Bhutan	H5N1	Chicken
India	H5N1, H5N8, H9N2, H4N6, H11N1	Chicken, duck, turkey
Maldives	-	-
Nepal	H5N1	Chicken, duck
Pakistan	H5N1, H7N3, H9N2, H3N1	Chicken, duck, geese, peacock, pigeon, pheasant, swan
Sri Lanka	-	-

**Table 6:** Summary of reported human cases of H5N1 virus in South Asia.

Country	Year of Detection							Total Cases	Total Deaths
	2007	2008	2011	2012	2013	2015	2019		
Bangladesh	-	1	2	3	1	1	-	8	1
Pakistan	3	-	-	-	-	-	-	3	1
Nepal	-	-	-	-	-	-	1	1	1
